# Optimization of ultrasonic extraction of polysaccharides from *Ziziphus jujuba* Mill. by response surface methodology

**DOI:** 10.1186/1752-153X-7-160

**Published:** 2013-09-23

**Authors:** Chenling Qu, Songcheng Yu, Li Luo, Yan Zhao, Yawei Huang

**Affiliations:** 1 College of Grain Oil and Food Science, Henan University of Technology, Zhengzhou 450052, China; 2 College of Public Health, Zhengzhou University, Zhengzhou 450001, China; 3 Changge Bureau of Quality and Technical Supervision, Changge 461500, China

**Keywords:** Ultrasonic extraction, Response surface methodology (RSM), Polysaccharides, *Ziziphus jujuba* Mill., Optimization

## Abstract

**Background:**

*Ziziphus jujuba* Mill. is nutritious and used as food and medicine for more than two thousand years. It has many pharmacological effects, such as elimination of fatigue, dilation of blood vessels, etc. The polysaccharide in it is one of the bioactive substances. In this paper, the ultrasonic extraction effects on the yield and activity of polysaccharide were studied.

**Results:**

The optimum ultrasonic extraction conditions were investigated based on a Box-Behnken statistical experimental design. Response surface methodology (RSM) of three factors (ultrasonic power, extraction time and extraction temperature) and three levels was employed to optimize the yield and the antioxidant activity of the polysaccharides. The experimental data were fitted to quadratic response surface models using multiple regression analysis. The best extraction conditions were 120 W, 15 min. and 55°C for highest yield, and 80 W, 15 min. and 40°C for highest hydroxyl radical scavenging activity.

**Conclusion:**

The study showed that high ultrasonic power was good for obtaining high yield but bad for keeping the antioxidant activity of the polysaccharides.

## Introduction

*Ziziphus jujuba* Mill. is a native plant of China and belongs to the genus *Ziziphus* Mill. (Rhamnaceae) [1]. Its fruits have been used in traditional Chinese medicine for more than two thousand years. The bioactivities of the polysaccharides in *Ziziphus jujuba* Mill. have been reported, such as immunobiological activities [1-4] and antioxidant activities [5].

Research reports revealed that the bioactivities of polysaccharides in *Ziziphus jujuba* Mill. were related to their structures. Chang *et al.*[5] isolated one neutral polysaccharide fraction (ZJPN) and three acidic polysaccharide fractions (ZJPa1, ZJPa2 and ZJPa3). Gas chromatography (GC) analysis revealed that six monosaccharides, namely, rhamnose, arabinose, xylose, mannose, glucose and galactose were present in the polysaccharide fractions. All four polysaccharide fractions were found to be more effective at scavenging superoxide anion radicals than hydroxyl radicals, while the acidic polysaccharides showed a more pronounced effect at chelating ferrous ion [5]. Zhao *et al.*[2] obtained a fraction, Ju-B-7, which could stimulate spleen cell proliferation and had a molecular mass of over 2000 kDa. This isolated polysaccaride was mainly composed of *α*-1,4-linked d-galactopyranosyluronic acid and 1,2-linked l-rhamnose at a molar ratio of 8.1:1.

Ultrasonic extraction was widely employed to extract polysaccharides from plant material due to its high extraction efficiency [6-9]. However, ultrasonicaion can change the structures of the polysaccharides to some extent [10]. In this paper, the effects of ultrasonic power, extraction time, extraction temperature on the yield and the antioxidant activity of water soluble polysaccharides of *Ziziphus jujuba* Mill. were investigated by response surface methodology (RSM).

RSM is an effective statistical technique, which is used to find optimum processing parameters [11-13]. It has been used to optimize the polysaccharides extraction process variables and the interactions of these variables [14-18]. In the present study, a three-variable, three-level Box–Behnken design (BBD) [19-25] was used to optimize the extraction conditions for ultrasonic extraction of water soluble polysaccharides in *Ziziphus jujuba* Mill.

## Experimental

### Chemicals and instruments

*Ziziphus jujuba* Mill., which grew in Xinjiang province (China) was purchased from a local shop in Zhengzhou, China. All reagents used in this study were of analytical grade. Anhydrous ethanol, 95% ethanol and acetone were obtained from Tianli Corporation (Tianjin, China). Ferrous sulfate (FeSO_4_), salicylic acid and petroleum ether were purchased from Kermel Corporation (Tianjin, China). Hydrogen peroxide (H_2_O_2_) was obtained from Haohua Corporation (Luoyang, China). Deionized water used in the experiments was purified by a Milli-Q system (Millipore Corporation, USA).

KQ5200DE ultrasonic cleaner, which can control ultrasonic temperature, power and time, was supplied by Kunshan Corporation (Shanghai, China). RE-52A rotary evaporator (Yarong Corporation, Shanghai, China) and 752 UV–vis spectrophotometer (Jinghua Corporation, Shanghai, China) were also employed in the experiments.

### Extraction procedure

The fruit of *Ziziphus jujuba* Mill. was first peeled, then the kernel was removed. The obtained pulp was dried at 40°C. The dried sample was extracted in a Soxhlet apparatus, first with petroleum ether, and afterwards with 80% ethanol twice, to remove some colored materials, monosaccharides, oligosaccharides, and small molecular weight materials. The organic solvent was evaporated to yield a dried extracted powder.

5.0 g of the Soxhlet-extracted powder was placed into a beaker with 100 g water. The powder was ultrasonically extracted for different time at varied extraction temperatures and power levels. Then the extraction solution was centrifuged for 15 min. at 4000 rpm. The supernatant was collected concentrated, and treated with 95% ethanol successively; the mixture was stored in a refrigerator at 4°C for 12 h. Afterwards, the obtained mixture was filtrated and the precipitate was successively washed by 95% ethanol, anhydrous ethanol and acetone. The washed precipitant, which was the crude polysaccharides, was dried at 40°C. The crude polysaccharides yield (%) was then calculated according to the following equation:

(1) $$\begin{array}{c} \mathrm{Polysaccharides}\;\mathrm{yield}\;\left(\%\right)\;=\\ \left(\mathrm{Polysaccharides}\;\mathrm{weight}/\mathrm{Soxhlet}‒\mathrm{extracted}\;\mathrm{powder}\;\mathrm{weight}\right)\times 100\% \end{array}$$

### Antioxidant activity

The hydroxyl radical scavenging activity of water soluble polysaccharides in *Ziziphus jujuba* Mill. was investigated by the following method. Approximately 2 mL of 1.8 mmol·L^-1^ FeSO_4_ and 1.5 mL of 1.8 mmol·L^-1^ salicylic acid were added into a tube and mixed. Then 1 mL of 3 mg·mL^-1^ polysaccharides solution was added along with 1 mL of 0.3% H_2_O_2_ and mixed to initiate the reaction. The tube was put into a 37°C water bath for 30 min.; afterwards, the UV–vis absorbance at 510 nm was recorded. 1 mL of water was used instead of 1 mL of 3 mg·mL^-1^ polysaccharides solution and other steps were same as polysaccharide sample to obtain the absorbance of the control. The hydroxyl radical scavenging activity of the polysaccharides was calculated using the following equation:

(2) $$\begin{array}{l} \mathrm{Scavenging}\;\mathrm{activity}\;\left(\%\right)\\ \;=\;\left[1‒\left(A_{\mathrm{sample}}/A_{\mathrm{control}}\right)\right]\times 100\% \end{array}$$

### Design of experiments

On the basis of single factor experiment, RSM was performed on the experimental data using a commercial statistical package, Design-Expert trial version 8.0.5 (Statease Inc., Minneapolis, USA) [26-28]. As shown in Table 1, a Box–Behnken design (BBD) with three independent variables, including ultrasonic power (X_1_), extraction time (X_2_), and extraction temperature (X_3_), was used for the optimization. On the basis of single factor experiments of ultrasonic extraction, three levels were coded as +1, 0, and −1 for high, intermediate and low values, respectively. The response functions were yield and hydroxyl radical scavenging activity of polysaccharides. The form of quadratic response model was as follows:

(3) $${Y=\beta }_{0}+\sum_{i‒1}^{3}\beta_{i}X_{i}+\sum_{i‒1}^{3}\beta_{\mathrm{ii}}X_{i}^{2}+\sum_{i‒1}^{2}\sum_{j‒i+1}^{3}\beta_{\mathrm{ij}}X_{i}X_{j}$$

where Y was the response variable, and *β*_o_, *β*_i_, *β*_ii_, and *β*_ij_, were the regression coefficients for the response surface model. *X*_i_ and *X*_j_ were the independent variables.

**Table 1:** **Box**–**Behnken design and the response values for yield and hydroxyl radical scavenging activity of polysaccharides**

**Run**	**X**_**1**_: **Ultrasonic Power (W)**	**X**_**2**_: **Extraction time (min)**	**X**_**3**_: **Extraction temperature (°C)**	**Yield of polysaccharides (%)**		**Hydroxyl radical scavenging activity of polysaccharides (%)**	
				**Actual values**	**Predicted values**	**Actual values**	**Predicted values**
1	120 (+1)	15 (+1)	50 (0)	4.44	4.53	35.61	35.66
2	80 (−1)	5 (−1)	50 (0)	3.26	3.17	49.36	49.31
3	100 (0)	10 (0)	50 (0)	3.98	4.03	50.14	50.37
4	100 (0)	10 (0)	50 (0)	3.96	4.03	48.35	50.37
5	100 (0)	10 (0)	50 (0)	4.06	4.03	51.65	50.37
6	100 (0)	5 (−1)	60 (+1)	3.56	3.59	38.43	36.73
7	120 (+1)	5 (−1)	50 (0)	3.80	3.82	39.86	41.42
8	80 (−1)	10 (0)	40 (−1)	2.96	3.01	65.65	65.51
9	100 (0)	10 (0)	50 (0)	4.12	4.03	51.26	50.37
10	100 (0)	15 (+1)	60 (+1)	4.16	4.12	41.65	41.46
11	120 (+1)	10 (0)	40 (−1)	3.82	3.76	47.52	45.77
12	80 (−1)	15 (+1)	50 (0)	3.58	3.56	65.16	63.60
13	100 (0)	10 (0)	50 (0)	4.05	4.03	50.45	50.37
14	100 (0)	15 (+1)	40 (−1)	3.68	3.65	51.68	53.38
15	100 (0)	5 (−1)	40 (−1)	3.04	3.08	49.36	49.56
16	80 (−1)	10 (0)	60 (+1)	3.40	3.45	49.56	51.31
17	120 (+1)	10 (0)	60 (+1)	4.36	4.31	35.08	35.22

### Statistical analyses

Design-Expert trial version 8.0.5 (Statease Inc., Minneapolis, USA) was used to statistically analyze the experimental data. The significant terms in the model were found by analysis of variance (ANOVA) for each response. The significances of all terms in the polynomial were considered statistically different when P < 0.05. The adequacy of model was checked by accounting for the coefficient of determination (R^2^) and adjusted-R^2^ (R^2^_adj_).

## Results and discussion

### Statistical analysis and the model fitting

Multiple regression analysis of the experimental data afforded the following quadratic response surface models for predicting polysaccharide yield (Y_yield_) and hydroxyl radical scavenging activity (Y_activity_) based on the values of the ultrasonic extraction parameters (*i.e.,* X_1_, X_2_ and X_3_):

(4) $$\begin{array}{l} Y_{\mathrm{yield}}=-8.99400+0.065625X_{1}+0.10060X_{2}+0.29375X_{3}\\ \;+8\times 10^{-4}X_{1}X_{2}+1.25\times 10^{-4}X_{1}X_{3}-2\times 10^{-4}X_{2}X_{3}\\ \;-2.9875\times 10^{-4}X_{1}^{2}-5.78\times 10^{-3}X_{2}^{2}-2.795\times 10^{-3}X_{3}^{2} \end{array}$$

(5) $$\begin{array}{l} Y_{\mathrm{activity}}=59.72625-0.49975X_{1}+8.03275X_{2}+0.44762X_{3}\\ \;-0.050125X_{1}X_{2}+4.5625\times 10^{-3}X_{1}X_{3}+4.5\times \\ \;10^{-3}X_{2}X_{3}+1.625\times 10^{-3}X_{1}^{2}-0.1409X_{2}^{2}\\ \;-0.015675X_{3}^{2} \end{array}$$

In these equations, X_1_, X_2_ and X_3_ were the values of extraction parameters, ultrasonic power (W), extracting time (min.) and extraction temperature (°C), respectively. The variables, experimental data and predicted data are shown in Table 1.

The fitted quadratic surface models for yield and hydroxyl radical scavenging activity of the polysaccharides by ANOVA are shown in Tables 2 and 3, respectively. The quadratic regression model of yield of polysaccharides in Table 2 showed the coefficient of determination coefficient, R^2^, value was 0.9837, while the value of the adjusted coefficient of determination coefficient, R^2^_adj_, was 0.9628, indicating a high degree of correlation between the observed and predicted values. The lower the coefficient of variation (CV), the smaller the residuals were relative to the predicted value. A low CV of 2.21% suggested a good precision and higher reliability of the models to predict experimental results. The “lack-of-fit F-value” of 2.54 implied that the lack-of-fit was not significant relative to the pure error. There was a 19.44% chance that a “lack-of-fit F-value” this large could occur due to noise, which indicated that the model equation was adequate for predicting the yield of polysaccharides. Values of P-value less than 0.05 indicated that the model terms were significant (at the 95% level).

**Table 2:** **Analysis of variance for the** fi**tted quadratic polynomial model of polysaccharides yield**

**Source**	**Sum of squares**	**Degree of freedom**	**Mean square**	***F-*Value**	***P***-**value**
					**Prob > F**
Model	2.94	9	0.33	47.07	<0.0001
X_1_	1.30	1	1.30	186.60	<0.0001
X_2_	0.61	1	0.61	87.10	<0.0001
X_3_	0.49	1	0.49	70.55	<0.0001
X_1_ X_2_	0.026	1	0.026	3.69	0.0964
X_1_ X_3_	2.5×10^-3^	1	2.5×10^-3^	0.36	0.5674
X_2_ X_3_	4.0×10^-4^	1	4.0×10^-4^	0.058	0.8172
X_1_^2^	0.060	1	0.060	8.66	0.0216
X_2_^2^	0.088	1	0.088	12.66	0.0092
X_3_^2^	0.33	1	0.33	47.36	0.0002
Residual	0.049	7	6.946×10^-3^		
Lack of Fit	0.032	3	0.011	2.54	0.1944
Pure Error	0.017	4	4.18×10^-3^		
Cor Total	2.99	16			
	R^2^=0.9837 R^2^_adj_=0.9628 CV=2.21%				

**Table 3:** **Analysis of variance for the** fi**tted quadratic polynomial model of hydroxyl radical scavenging activity of polysaccharides**

**Source**	**Sum of squares**	**Degree of freedom**	**Mean square**	***F-*Value**	***P***-**value**
					**Prob > F**
Model	1154.38	9	128.26	38.82	<0.0001
X_1_	641.89	1	641.89	191.75	<0.0001
X_2_	36.51	1	36.51	10.91	0.0131
X_3_	306.16	1	306.16	91.46	<0.0001
X_1_ X_2_	100.50	1	100.50	30.02	0.0009
X_1_ X_3_	3.33	1	3.33	0.99	0.3518
X_2_ X_3_	0.20	1	0.20	0.060	0.8128
X_1_^2^	1.78	1	1.78	0.53	0.4897
X_2_^2^	52.24	1	52.24	15.61	0.0055
X_3_^2^	10.35	1	10.35	3.09	0.1222
Residual	23.43	7	3.35		
Lack of Fit	16.86	3	5.62	3.42	0.1328
Pure Error	6.57	4	1.64		
Cor Total	1177.82	16			
	R^2^=0.9801 R^2^_adj_=0.9545 CV=3.79%				

Table 3 showed the quadratic regression model of hydroxyl radical scavenging activity of the polysaccharides. It can be seen that R^2^ was 0.9801 and R^2^_adj_ was 0.9545, indicating a high degree of correlation between the observed and predicted values. The coefficient of variation was low (CV=3.79%), indicating a high degree of precision and reliability of the experimental values. F-value and P-value of the lack-of-fit were 3.42 and 0.1328, respectively, which implied that it was not significant; there was a 13.28% chance that this lack-of-fit was due to noise. It can be seen from Table 3 that the three independent variables (X_1_, X_2_ and X_3_), one quadratic term (X_2_^2^), and the interaction between X_1_ and X_2_ significantly affected the hydroxyl radical scavenging activity of the polysaccharides.

### Analysis of response surface plot

**Figure 1:**
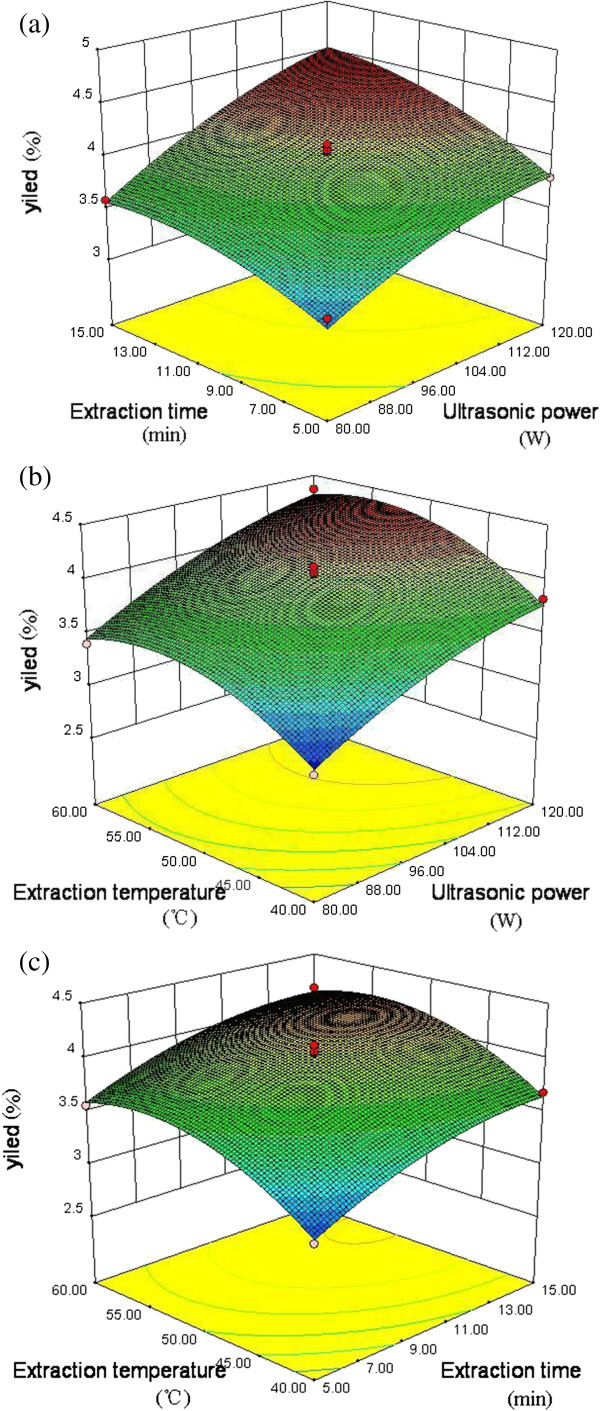
**Response surface plot results on the yield of polysaccharides. (a)** Response surface plot of ultrasonic power and extraction time, and their mutual interactions on the yield of polysaccharides. **(b)** Response surface plot of ultrasonic power and extraction temperature, and their mutual interactions on the yield of polysaccharides. **(c)** Response surface plot of extraction time and extraction temperature, and their mutual interactions on the yield of polysaccharides.

**Figure 2:**
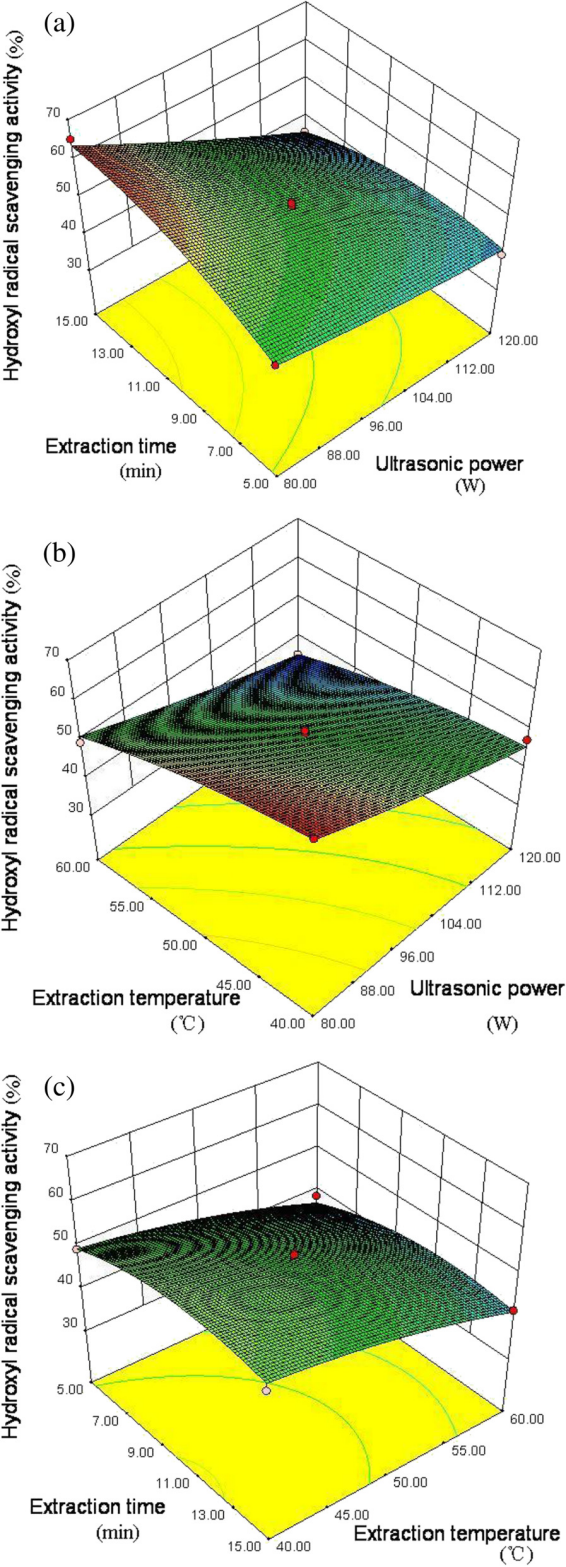
**Response surface plot results on hydroxyl radical scavenging activity of polysaccharides. (a)** Response surface plot of ultrasonic power and extraction time, and their mutual interactions on hydroxyl radical scavenging activity of polysaccharides. **(b)** Response surface plot of ultrasonic power and extraction temperature, and their mutual interactions on hydroxyl radical scavenging activity of polysaccharides. **(c)** Response surface plot of extraction time and extraction temperature, and their mutual interactions on hydroxyl radical scavenging activity of polysaccharides.

Response surface models were plotted to study the effects of parameter variables (ultrasonic power, extraction time and extraction temperature) and their interactions on yield (Figure 1) and hydroxyl radical scavenging activity (Figure 2) of the polysaccharides. When two variables within the experimental range were displayed in three-dimensional surface plots, the third variable was kept constant at the intermediate level (*i.e*., 0).

As shown in Figure 1a, when the extraction temperature (X_3_) was fixed at 0 level, the yield increased as the ultrasonic power (X_1_) and extraction time (X_2_) increased. Figure 1b showed the effects of ultrasonic power (X_1_) and extraction temperature (X_3_) on the yield of polysaccharides. The yield increased with the increase of ultrasonic power. The yield was positively correlated with the extraction temperature when temperature was lower than 55°C and was negatively correlated when temperature was higher than 55°C. The interactions between extraction time (X_2_) and extraction temperature (X_3_), when ultrasonic power (X_1_) was fixed at 0 level, were displayed in Figure 1c. The yield increased with the extraction time.

Figure 2 showed the ultrasonic parameter variables (ultrasonic power, extraction time and extraction temperature) and their interactions on hydroxyl radical scavenging activity of polysaccharides. Ultrasonic power (X_1_) and extraction temperature (X_3_) both had a negative impact on the activity. Nevertheless, longer extraction times led to an increase of the activity. Therefore, low extraction temperature and low ultrasonic power were advantageous to the hydroxyl radical scavenging activity of polysaccharides.

### Optimization of extracting parameters and validation of the model

In Table 4, the optimal ultrasonic extraction condition for obtaining maximal yield of polysaccharides predicted by the quadratic model was as follows: ultrasonic power of 120 W, extraction time of 15 min. and extraction temperature of 54.69°C. The predicted yield of polysaccharides at the optimal extraction condition was 4.59%. In order to facilitate the extraction process, the optimal condition was modified as follows: ultrasonic power of 120 W, extraction time of 15 min. and extraction temperature of 55°C. The actual experimental yield under these conditions was 4.47%, which was in agreement with the predicted model value.

**Table 4:** **Optimum conditions**, **and the predicted and experimental values of response**

	**Ultrasonic power (W)**	**Extraction time (min)**	**Extraction temperature (°C)**	**Yield of polysaccharides (%)**	**Hydroxyl radical scavenging activity of polysaccharides (%)**
Optimum condition for yield (predicted)	120	15	54.69	4.59	32.75
Modified condition for yield (actual)	120	15	55	4.47	30.94
Optimum condition for activity (predicted)	80	14.91	40	3.07	68.91
Modified condition for activity (actual)	80	15	40	2.91	67.30

The optimal predicted extraction condition for achieving the highest hydroxyl radical scavenging activity of 68.91% was ultrasonic power of 80 W, extraction time of 14.91 min. and extraction temperature of 40°C. For practical implementation, the extraction condition was modified as ultrasonic power of 80 W, extraction time of 15 min. and extraction temperature of 40°C. Using these parameters, the hydroxyl radical scavenging activity was 67.30%, which was close to the maximum predicted by the response surface model (Table 4).

Table 4 also displayed that the hydroxyl radical scavenging activity of the polysaccharides under the optimal condition for highest yield (ultrasonic power of 120 W, extraction time of 15 min. and extraction temperature of 54.69°C) was predicted as 32.75% by the quadratic response surface model (Eq. [5]), and the activity obtained at the experiment condition (ultrasonic power of 120 W, extraction time of 15 min. and extraction temperature of 55°C) was 30.94%. At the same time, the yield of polysaccharides under the optimal condition for best hydroxyl radical scavenging activity of polysaccharides (ultrasonic power of 80 W, extraction time of 14.91 min. and extraction temperature of 40°C) was predicted as 3.07% by Equation [4]. The yield in the modified condition (ultrasonic power of 80 W, extraction time of 15 min. and extraction temperature of 40°C) was 2.91%.

These data suggested that the extraction conditions for obtaining high yield of polysaccharides were not suitable for obtaining good hydroxyl radical scavenging activity, and that the optimal conditions for achieving high hydroxyl radical scavenging activity could not be applied to obtain high yield of polysaccharides. High ultrasonic power was advantageous to yield and adverse to activity, and low extraction temperature was more favorable for high radical scavenging activity. Extraction time 15 min. was good to both the yield and the activity.

## Conclusion

The results indicated that the optimum extraction conditions of polysaccharides for obtaining highest yield and highest radical scavenging activity were quite different. Ultrasonic power played an important role in ultrasonic extraction.

Therefore, we should consider not only the high yield but also the sacrificed radical scavenging activity of the polysaccharides during the extraction process.

## Abbreviations

RSM: Response surface methodology; GC: Gas chromatography; BBD: Box–Behnken design; ANOVA: Analysis of variance; Yyield: Polysaccharide yield; Yactivity: Hydroxyl radical scavenging activity; CV: Coefficient of variation.

## Competing interests

The authors declare that they have no competing interests.

## Authors’ contributions

CQ participated in the design of the study and performed the statistical analysis. CQ and YZ participated in the sequence alignment and drafted the manuscript. SY and LL carried out the experiments. YH participated in its design and coordination and helped to draft the manuscript. All authors read and approved the final manuscript.
